# Dimethylarginine Dimethylaminohydrolase 1 (DDAH1)-Arginine Metabolic Axis Is Independently Associated With Alzheimer’s Disease Risk in the UK Biobank

**DOI:** 10.7759/cureus.114415

**Published:** 2026-08-12

**Authors:** Steven Lehrer, Peter Rheinstein

**Affiliations:** 1 Radiation Oncology, Icahn School of Medicine at Mount Sinai, New York, USA; 2 Family Medicine, Severn Health Solutions, Severna Park, USA

**Keywords:** alzheimer’s disease, arginine metabolism, ddah1, nitric oxide signaling, uk biobank

## Abstract

Background

Altered arginine metabolism and nitric oxide (NO) signaling have been implicated in Alzheimer’s disease (AD), but the role of dimethylarginine dimethylaminohydrolase 1 (DDAH1), a regulator of nitric oxide bioavailability, remains unclear. We investigated whether the DDAH1-arginine metabolic axis is independently associated with AD risk in the UK Biobank (UKB).

Methods

We analyzed 50,988 UK Biobank participants with available proteomic, metabolomic, clinical, genetic, and neuroimaging data. Plasma DDAH1 levels were measured using the Olink Explore 3072 platform (Olink Proteomics AB, Uppsala, Sweden). Associations between DDAH1, circulating arginine, and AD were evaluated using multivariable logistic regression adjusted for age, sex, education, APOE ε4 dosage, and genetic principal components. Interaction analyses assessed whether DDAH1 modified the association between arginine and AD risk. Structural MRI phenotypes were examined using multivariable linear regression.

Results

DDAH1 levels were significantly elevated in AD cases in unadjusted analyses (p = 0.001). Higher systemic arginine was independently associated with lower AD risk (odds ratio {OR} = 0.604; p = 0.0033). A significant DDAH1-arginine interaction persisted after multivariable adjustment (OR = 1.431; p = 0.000108), indicating that the association between arginine and AD differed according to DDAH1 expression. Most MRI phenotypes were not significantly associated with DDAH1, although reduced hippocampal volume was observed in a neuroimaging subset.

Conclusions

The DDAH1-arginine metabolic axis is independently associated with AD risk and may represent a biologically relevant pathway linking nitric oxide dysregulation to neurodegeneration.

## Introduction

Alterations in arginine metabolism have emerged as promising biomarkers and potential mechanistic contributors to neurodegeneration, particularly Alzheimer’s disease (AD) [1-4]. Arginine serves as the substrate for nitric oxide synthase (NOS), generating nitric oxide (NO), a signaling molecule essential for cerebrovascular health, synaptic plasticity, and neuroimmune regulation [5-7]. Disruptions to NO bioavailability are increasingly recognized as important in AD, where endothelial dysfunction, neuroinflammation, and impaired metabolic signaling converge [8]. Dimethylargininase, a zinc protein involved in the regulation of nitric oxide synthase, is specifically elevated in neurons displaying cytoskeletal abnormalities and oxidative stress in AD, while none of this enzyme was found in neurons in age-matched control cases [9].

A central regulator of arginine-NO homeostasis is dimethylarginine dimethylaminohydrolase 1 (DDAH1), the enzyme responsible for degrading asymmetric dimethylarginine (ADMA), the endogenous inhibitor of NOS [10]. Reduced DDAH1 activity increases ADMA levels and thereby suppresses NO production, potentially impairing endothelial function and neuronal resilience. Although dysregulated ADMA-NO signaling has been implicated in vascular and metabolic disease, its role in AD pathophysiology remains poorly defined [11,12]. While DDAH1 dysregulation may be a biochemical feature of AD, whether DDAH1 is an independent marker of AD risk, or whether it reflects age-related physiological changes that correlate with but do not cause disease, remains unclear.

Large population-based cohorts such as the UK Biobank (UKB) allow for the deeper evaluation of these questions through the integration of proteomics, cognitive testing, genetics, and structural neuroimaging. UKB contains Olink proteomic measurements for DDAH1, fluid intelligence testing as a marker of cognitive performance, and high-resolution brain MRI for a subset of participants [13]. These multimodal data enable the examination of (1) the relationship between DDAH1 and AD risk, (2) whether DDAH1 variation associates with structural brain changes in regions vulnerable to AD pathology, and (3) whether DDAH1 relates to cognitive performance independent of age and sex. Furthermore, the availability of genome-wide principal components allows adjustment for population structure, improving the interpretability of biomarker-phenotype associations [14].

Given the biological importance of NO signaling in vascular and neuroimmune pathways and the preliminary evidence that DDAH1 distinguishes AD cases from controls, we investigated DDAH1 as an arginine-related biomarker of AD in UKB. We examined its associations with AD diagnosis, cognitive performance, and MRI-derived structural measures, adjusting for age, sex, and genetic principal components. We additionally evaluated whether DDAH1 contributes to AD risk beyond established demographic and cognitive factors and whether DDAH1 variation corresponds to macrostructural brain differences in regions implicated in early AD pathology, including the parahippocampal gyrus, entorhinal cortex, amygdala, and deep white matter hyperintensities. This multimodal approach provides a comprehensive assessment of the role of DDAH1 in AD-related biological pathways and evaluates its potential utility as a biomarker for AD risk or disease-related brain changes.

The primary objective of this study was to determine whether the DDAH1-arginine metabolic axis, including the interaction between circulating DDAH1 and arginine, is associated with Alzheimer’s disease independently of established demographic and genetic risk factors. Secondary objectives were to evaluate associations between DDAH1 and AD-related structural brain MRI phenotypes and to explore whether these findings provide neurobiological support for the involvement of the arginine-nitric oxide pathway. We hypothesized that the DDAH1-arginine interaction would remain associated with AD after adjustment for age, sex, education, APOE ε4 dosage, and genetic population structure. The imaging analyses were considered secondary and exploratory.

## Materials and methods

Study population

We performed a cross-sectional analysis using data from the UK Biobank, a large population-based prospective cohort with linked clinical, genetic, proteomic, metabolomic, cognitive, and neuroimaging data. Participants were included if they had available Olink proteomic measurements, metabolomic arginine measurements, Alzheimer’s disease status, age, sex, and the first three genetic principal components (PC1-PC3). The primary analytic cohort included 50,988 participants with complete proteomic, metabolomic, and clinical data. Alzheimer’s disease cases were identified from UK Biobank diagnostic records, and all remaining participants without Alzheimer’s disease were classified as controls.

Missing data and participant selection

Analyses used a complete-case approach. Participants lacking data required for a particular model were excluded from that analysis; missing values were not imputed. Consequently, sample size varied across analyses according to data availability, particularly for the neuroimaging analyses. The primary analytic cohort comprised 50,988 participants with the data required for the principal proteomic, metabolomic, and clinical analyses, including 271 AD cases and 50,717 controls. The neuroimaging analysis was restricted to participants with the corresponding MRI-derived phenotype available.

Study objectives

The prespecified primary analysis evaluated the association of systemic arginine, circulating DDAH1, and their interaction with AD status. Secondary analyses evaluated associations between DDAH1 and selected structural MRI phenotypes relevant to AD. The MRI analyses were exploratory and were intended to assess whether the metabolic associations observed in the primary analysis were accompanied by detectable macrostructural brain differences.

Alzheimer’s disease status was determined from the UK Biobank diagnostic data available for the analysis. Participants recorded as having Alzheimer’s disease were classified as cases, and participants without a recorded Alzheimer’s disease diagnosis were classified as controls. The present analysis used the UK Biobank-derived diagnostic classification rather than independent adjudication or neuropathological confirmation.

Proteomic and metabolomic measurements

Plasma protein levels were measured using the Olink proteomics platform and expressed as normalized protein expression values. The primary protein of interest was dimethylarginine dimethylaminohydrolase 1 (DDAH1). Additional arginine-related proteins, including PADI2 and PADI4, were evaluated in preliminary case-control comparisons. Circulating arginine was analyzed as a systemic metabolomic measure. For regression analyses and interaction modeling, DDAH1 and arginine values were standardized as z-scores to allow effect estimates to be interpreted per standard deviation (SD) increase.

Covariates

Covariates included age at recruitment, sex, years of education, APOE ε4 allele dosage, and the first three genetic principal components (PC1-PC3). Genetic principal components were included to reduce confounding by population structure. Primary multivariable models were adjusted for age, sex, and genetic principal components. Fully adjusted models additionally included years of education and APOE ε4 allele dosage.

Alzheimer’s disease case-control comparisons

Mean DDAH1, PADI2, and PADI4 levels were compared between Alzheimer’s disease cases and controls using two-sample t-tests. Results were visualized using bar plots showing mean normalized protein expression with standard error bars. These analyses were considered preliminary screening analyses to identify arginine-related proteins most strongly associated with Alzheimer’s disease status.

Logistic regression analysis

Associations between DDAH1, arginine, and Alzheimer’s disease were evaluated using multivariable logistic regression. Alzheimer’s disease status was modeled as the binary dependent variable. Independent variables included standardized DDAH1, standardized arginine, and their multiplicative interaction term. Models were adjusted for age, sex, the first three genetic principal components (PC1-PC3), years of education, and APOE ε4 allele dosage. Odds ratios (ORs) and 95% confidence intervals (CIs) were calculated from model coefficients. The DDAH1-arginine interaction was used to test whether the association between systemic arginine and Alzheimer’s disease risk differed according to DDAH1 protein level.

Predicted probability modeling

To visualize the interaction between DDAH1 and arginine, predicted probabilities of Alzheimer’s disease were generated from the adjusted logistic regression model across the observed range of standardized arginine values. Predictions were plotted at three representative DDAH1 levels: low DDAH1, defined as -1 SD; mean DDAH1; and high DDAH1, defined as +1 SD. Predicted probabilities were adjusted for age, sex, years of education, the first three genetic principal components (PC1-PC3), and APOE ε4 allele dosage.

Brain MRI measures

A subset of UK Biobank participants had available structural brain MRI data. Four MRI-derived measures were selected because of their relevance to Alzheimer’s disease and neurodegeneration: the area of the left parahippocampal region, total volume of deep white matter hyperintensities, mean thickness of the left entorhinal cortex, and gray matter volume of the left amygdala. Associations between standardized DDAH1 and each MRI measure were tested using multivariable linear regression. Models were adjusted for age, sex, and genetic principal components.

MRI measures were preexisting UK Biobank imaging-derived phenotypes rather than investigator-assigned visual ratings. No manual image grading was performed for the present study; therefore, investigator blinding and inter-rater reliability assessments were not applicable.

Statistical analysis

Continuous variables were compared between Alzheimer’s disease cases and controls using Welch’s two-sample t-test, and categorical variables were compared using Pearson’s χ² test. Corresponding test statistics and two-sided P values were reported. Statistical significance was defined as two-sided P < 0.05. Categorical variables were summarized as frequency (percentage) and compared using Pearson’s χ² test. Multivariable associations with Alzheimer’s disease were evaluated using logistic regression, and adjusted odds ratios (ORs) with 95% confidence intervals (CIs) were estimated. Wald χ² statistics and two-sided P values were used to assess the statistical significance of individual regression coefficients. For analyses of MRI-derived continuous outcomes, multivariable linear regression was performed and regression coefficients, t-statistics, and two-sided P values were reported. All statistical tests were two-sided, and statistical significance was defined as P < 0.05.

No prospective sample-size calculation was performed because this was a secondary analysis of an existing cohort. The analytic sample was determined by the availability of the required UK Biobank proteomic, metabolomic, clinical, genetic, and, where applicable, imaging variables.

The DDAH1-arginine interaction constituted the primary hypothesis-driven analysis. Secondary biomarker screening and neuroimaging analyses were exploratory, and no formal adjustment for multiple comparisons was applied to these analyses. Accordingly, secondary findings should be interpreted as exploratory and hypothesis-generating.

Statistical analyses were performed using R version 4.5 (R Foundation for Statistical Computing, Vienna, Austria).

## Results

Participant characteristics and arginine-DDAH1 profiles

The final analysis included 50,988 participants from the UK Biobank with complete proteomic, metabolomic, and clinical data. In preliminary case-control comparisons, DDAH1 levels were significantly higher in individuals with Alzheimer’s disease (AD) compared to healthy controls (p = 0.001), though this association was heavily influenced by age (Table 1).

**Table 1:** Baseline characteristics of participants with and without Alzheimer’s disease (AD). Continuous variables are presented as mean ± standard deviation and categorical variables as number (%). Welch’s two-sample t-test was used for continuous variables and Pearson’s χ² test for categorical variables. All tests were two-sided, and statistical significance was defined as P < 0.05. Sample sizes vary because of missing data. Arginine measurements were available for 11,003 participants; other variables were available for 42,448-42,988 participants. DDAH1, dimethylarginine dimethylaminohydrolase 1; NPX, normalized protein expression

Characteristic	Total	AD cases	Controls	Test statistic	P value
Age at recruitment, years	56.7 ± 8.2	64.8 ± 5.1	56.7 ± 8.2	t = 24.19	<0.001
Male sex, n (%)	19,556 (45.5%)	103 (44.8%)	19,453 (45.5%)	χ² = 0.05	0.829
Arginine	0.062 ± 0.014	0.060 ± 0.014	0.062 ± 0.014	t = -1.00	0.324
DDAH1 protein, NPX	0.038 ± 0.510	0.127 ± 0.535	0.037 ± 0.510	t = 2.53	0.012
Years of education	14.7 ± 4.8	12.4 ± 5.1	14.7 ± 4.8	t = -6.53	<0.001
Genetic principal component 1	0.0001 ± 0.0017	-0.0001 ± 0.0014	0.0001 ± 0.0017	t = -2.03	0.043

**Figure 1:**
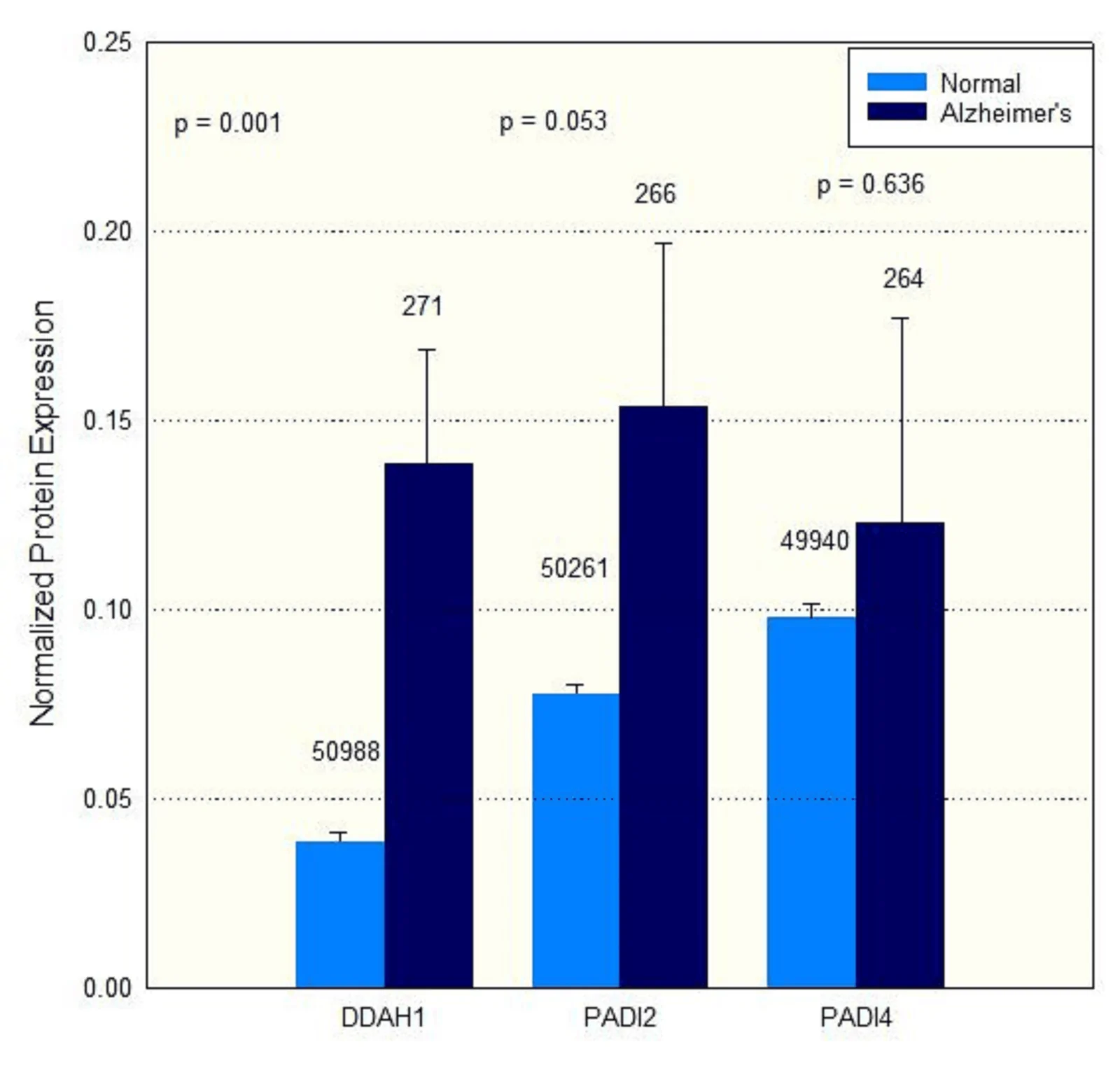
Group differences in arginine-related proteins (DDAH1, PADI2, and PADI4) in Alzheimer’s disease and controls. Plasma levels of DDAH1, PADI2, and PADI4 in participants with and without Alzheimer’s disease. Bars represent mean normalized protein expression (NPX) ± standard error (SE). Group comparisons were performed using Welch’s two-sample t-test. DDAH1 levels were significantly higher in participants with Alzheimer’s disease (Welch’s t = -3.07; two-sided P = 0.002). PADI2 showed a nonsignificant trend, whereas PADI4 did not differ significantly between groups. DDAH1: dimethylarginine dimethylaminohydrolase 1

In an initial analysis (Figure 1), we compared mean levels of three arginine-related proteins, DDAH1, PADI2, and PADI4, between individuals with Alzheimer’s disease (AD) and controls. DDAH1 demonstrated a statistically significant difference between groups (t = -3.22; p = 0.001), with higher values observed in AD cases (mean: 0.1399) compared to controls (mean: 0.0396). PADI2 showed a trend toward higher values in AD (t = -1.93; p = 0.053), whereas PADI4 showed no significant group difference (p = 0.636). These preliminary results suggested that DDAH1 may be the arginine-related biomarker most strongly associated with AD.

**Figure 2:**
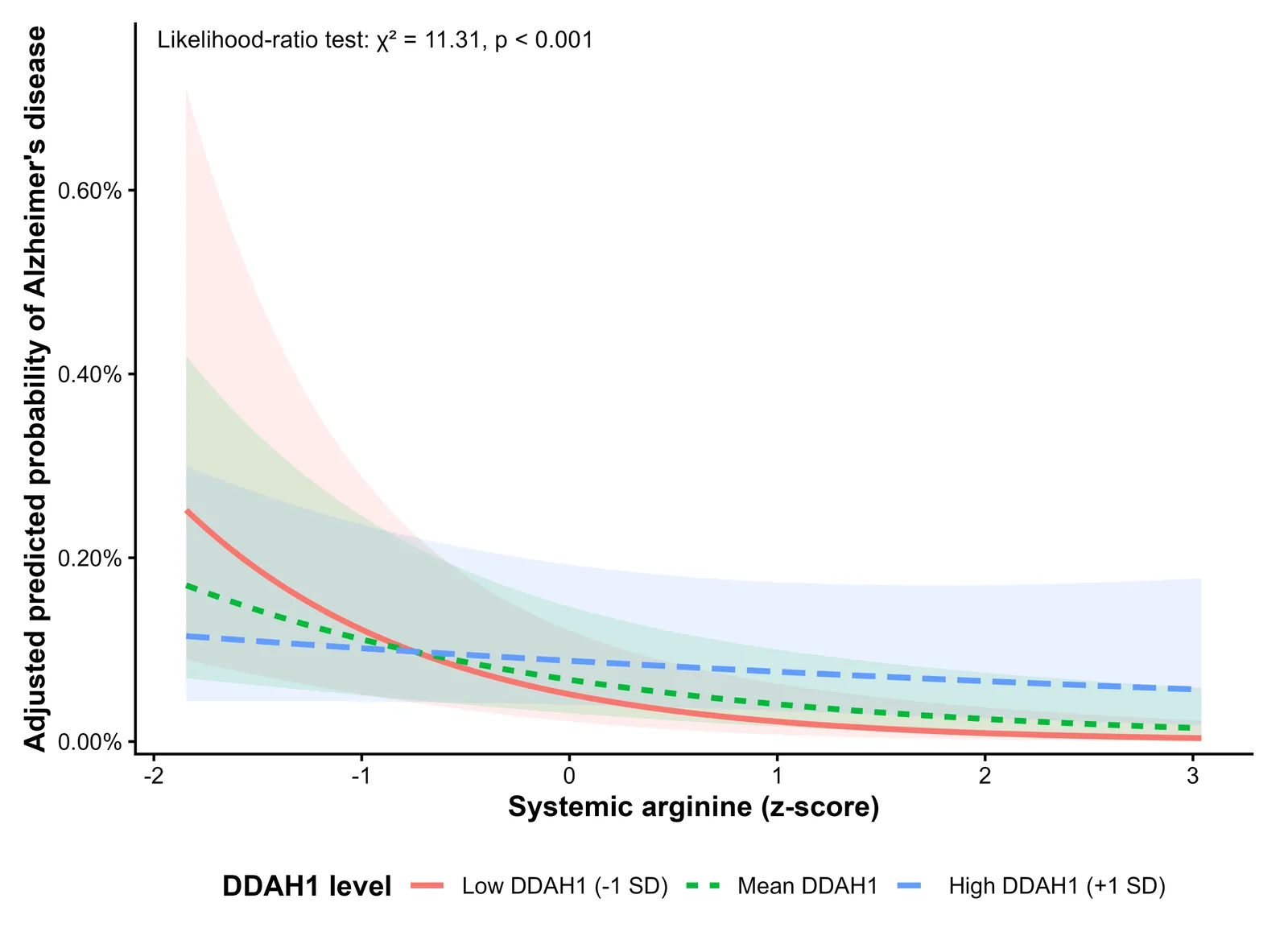
Association of the DDAH1-arginine interaction with Alzheimer’s disease (AD) probability. Predicted probability of AD diagnosis plotted as a function of systemic arginine concentration (z-score) at three representative levels of DDAH1 protein expression: high (+1 SD), mean, and low (-1 SD). A significant DDAH1-arginine interaction was observed (p = 0.000108), indicating that the association between arginine and predicted AD probability differed according to DDAH1 level. Probabilities are adjusted for age, sex, educational attainment, population structure (PC1-PC3), and APOE ε4 allele dosage. The interaction represents an observational association and does not establish a causal or therapeutic effect. DDAH1, dimethylarginine dimethylaminohydrolase 1; SD, standard deviation

Although preliminary case-control comparisons showed higher DDAH1 levels in participants with AD, the association was substantially attenuated after accounting for age and metabolic covariates. In the multivariable model, a significant DDAH1-arginine interaction was observed (p < 0.001), indicating that the association between circulating arginine and AD status differed according to DDAH1 level. Higher arginine was associated with lower predicted AD probability, although the magnitude of this association varied across DDAH1 levels (Figure 2). These observational findings are consistent with the involvement of the DDAH1-arginine axis but do not establish the underlying mechanism, the direction of effect, or whether altered DDAH1 represents a causal, compensatory, or secondary response. The results suggest that the neuroprotective potential of arginine, recently identified in preclinical models as a chemical chaperone capable of suppressing Abeta42 aggregation and neuroinflammation, is fundamentally dependent on DDAH1-mediated degradation of the NOS inhibitor ADMA [12]. Consequently, the observed elevation of DDAH1 in AD cases likely represents a localized homeostatic response to maintain NO bioavailability in a substrate-depleted environment, a mechanism that could be leveraged for the therapeutic repositioning of oral arginine.

Across all four structural brain MRI measures, DDAH1 was not significantly associated with regional brain structure (β range: -0.045 to +0.027 SD per SD DDAH1). As expected, age demonstrated negative associations with most MRI phenotypes, consistent with age-related atrophy, and sex differences aligned with known structural patterns, with men showing larger volumes for the area of the parahippocampal region (left hemisphere) and gray matter in the amygdala (left). Overall, these results indicate that DDAH1 levels are not linked to macrostructural brain changes in the imaging subset of the UK Biobank.

In the neuroimaging sub-cohort (N = 1,976), we examined whether the DDAH1-arginine axis predicted total hippocampal volume. While age and sex were dominant predictors of volume, DDAH1 expression was independently associated with reduced hippocampal volume (ß = -0.052; p = 0.016). However, unlike the clinical risk model, the DDAH1-arginine interaction did not reach statistical significance for macrostructural volume (p = 0.625), suggesting that the metabolic synergy observed in AD risk may manifest at molecular or microvascular levels before regional atrophy is detectable.

Multivariable modeling controlling for genetic and cognitive reserve confounders

To verify whether the synergistic association of the DDAH1-arginine axis was independent of established primary risk factors, multivariable logistic regression was performed adjusting for age, sex, population structure (PC1-PC3), years of education, and APOE ε4 allele dosage.

As expected, age (OR = 1.23; p < 0.001) and APOE ε4 carrier status were highly dominant predictors of AD risk. In the categorical model, heterozygous ε4 carriers demonstrated a 3.00-fold increase in the odds of AD (95% CI, 1.64-5.45; p = 0.000289), whereas homozygous ε4 carriers demonstrated an 8.27-fold increase (95% CI, 3.63-17.6; p < 0.001). Higher educational attainment conferred an independent protective effect (OR = 0.928 per year of education; p = 0.0093).

**Figure 3:**
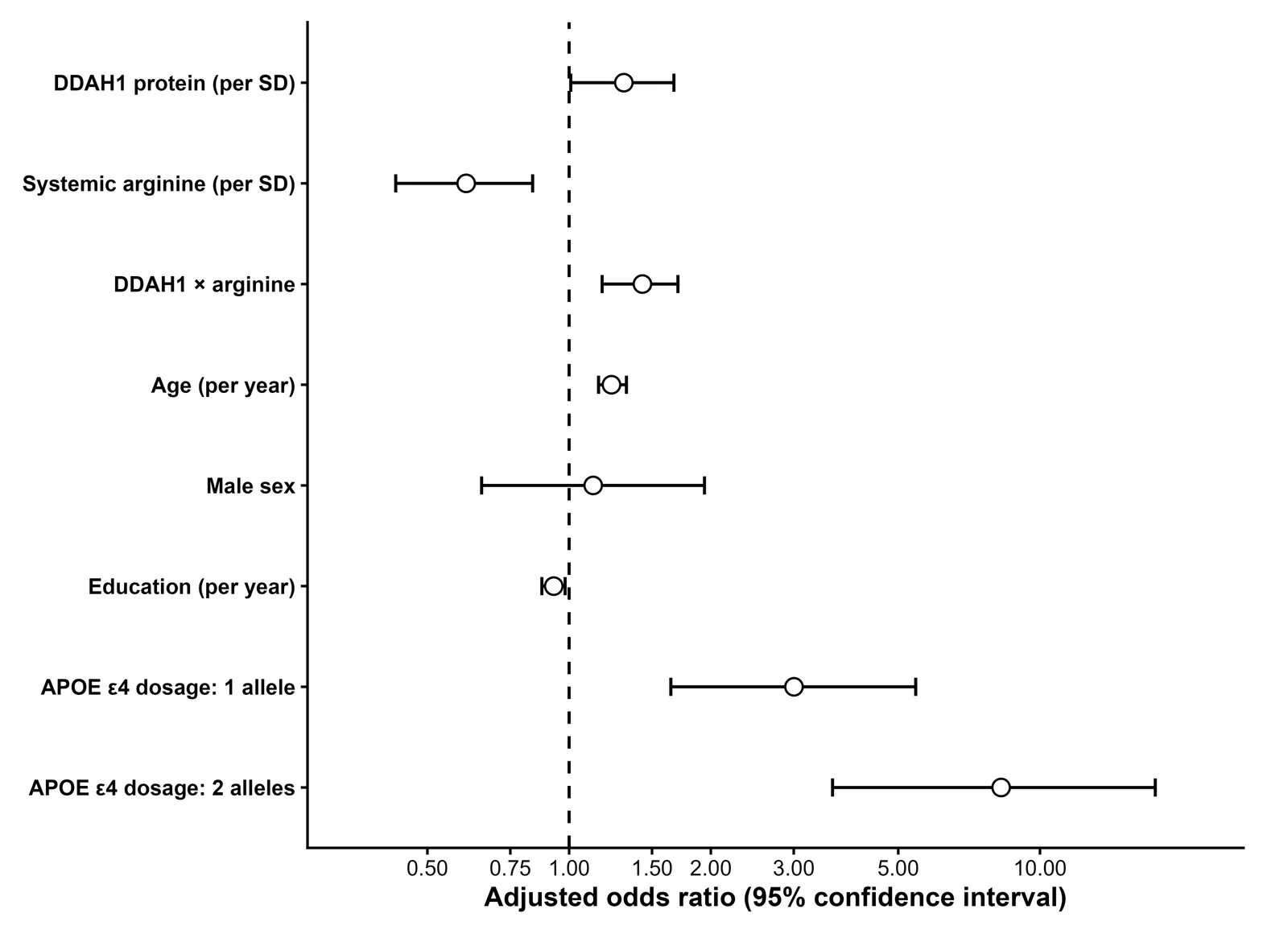
Multivariable odds ratios for Alzheimer’s disease risk factors. Forest plot of adjusted odds ratios (ORs) and 95% confidence intervals (CIs) from the multivariable logistic regression model evaluating factors associated with Alzheimer’s disease. The model included DDAH1, arginine, age, sex, genetic principal components (PC1-PC3), and the DDAH1-arginine interaction. Wald χ² statistics and corresponding two-sided P values for the principal predictors were as follows: DDAH1, Wald χ² = 2.11 and P = 0.147; arginine, Wald χ² = 7.62 and P = 0.006; age, Wald χ² = 46.7 and P < 0.001; sex, Wald χ² = 0.07 and P = 0.798; and the DDAH1-arginine interaction, Wald χ² = 11.0 and P < 0.001. Odds ratios are presented with 95% confidence intervals. DDAH1, dimethylarginine dimethylaminohydrolase 1; SD, standard deviation

Crucially, after accounting for these powerful background genetic and socioeconomic variances, systemic arginine levels maintained a significant protective main association (OR = 0.604; p = 0.0033). Most importantly, the synergistic interaction between DDAH1 and arginine remained highly significant (OR = 1.431; p = 0.000108). This statistical persistence suggests that the DDAH1-arginine axis may represent an additional metabolic dimension associated with AD risk independent of established demographic and genetic factors (Figure 3).

## Discussion

In this large, multimodal analysis of UK Biobank participants, circulating DDAH1 was the arginine-related protein most clearly associated with Alzheimer’s disease (AD) in preliminary case-control comparisons. The fully adjusted analyses, however, revealed a more complex relationship than a simple association between higher DDAH1 levels and disease. Higher circulating arginine was independently associated with lower odds of AD, whereas the significant DDAH1-arginine interaction indicated that the association between arginine and AD varied according to DDAH1 protein level. This interaction remained significant after adjustment for age, sex, educational attainment, APOE ε4 dosage, and genetic principal components. Thus, the principal finding is not that DDAH1 alone functions as an isolated AD biomarker but that DDAH1 and systemic arginine appear to operate as an interdependent metabolic axis associated with AD risk.

These findings are consistent with evidence that arginine metabolism is altered in dementia and may reflect both disease severity and structural brain changes. Targeted metabolomic studies have reported disturbances involving arginine, citrulline, ornithine, and metabolites related to nitric oxide production in patients with dementia [1]. More broadly, the dysregulation of arginase and competing arginine-consuming pathways has been implicated in neurodegenerative disease through effects on substrate availability, oxidative stress, immune activation, and cellular metabolism [2,3]. Experimental work in an AD mouse model further suggests that alterations in the plasma arginine metabolome can precede corresponding behavioral and cerebral metabolic abnormalities [4]. Our finding that higher systemic arginine was independently associated with lower AD risk therefore supports previous evidence that peripheral arginine metabolism may be biologically relevant rather than merely an epiphenomenon of established dementia.

One potential explanation for the inverse association between arginine and AD is improved nitric oxide availability. Arginine is the substrate for nitric oxide synthase, and nitric oxide has important roles in vascular tone, cerebral perfusion, endothelial integrity, synaptic plasticity, and neuroimmune regulation [5-7]. Endothelial nitric oxide deficiency has been shown experimentally to promote several features of AD pathology, including cerebral amyloid accumulation, microvascular dysfunction, and cognitive impairment [8]. Consequently, lower effective arginine availability could contribute to AD susceptibility through impaired neurovascular coupling and reduced capacity to maintain cerebral blood flow. Nevertheless, circulating arginine concentration cannot be assumed to directly represent nitric oxide production, because its utilization depends on nitric oxide synthase activity, competing arginase pathways, transport into cells, oxidative conditions, and endogenous nitric oxide synthase inhibitors. The observed association should therefore be interpreted as evidence implicating the broader arginine-nitric oxide system rather than as proof that increasing plasma arginine alone would prevent AD.

DDAH1 provides a plausible regulatory link within this system. DDAH1 degrades asymmetric dimethylarginine (ADMA), an endogenous inhibitor of nitric oxide synthase, and thereby contributes to the regulation of nitric oxide bioavailability [9]. In principle, greater DDAH1 activity should reduce ADMA-mediated inhibition and facilitate nitric oxide production when sufficient arginine is available. The significant interaction observed in the present study is compatible with the concept that the association between systemic arginine and AD depends partly on regulation of the ADMA-nitric oxide synthase pathway. The data do not, however, demonstrate that circulating DDAH1 protein is equivalent to tissue-specific enzymatic activity. Neither ADMA concentration nor nitric oxide production was measured directly. The biological interpretation of the interaction therefore remains provisional and should be tested in studies incorporating DDAH1 activity, ADMA, symmetric dimethylarginine, citrulline, ornithine, and direct indices of endothelial function.

The direction of the interaction also requires cautious interpretation. Although arginine showed an overall inverse association with AD, the interaction OR greater than 1 indicates that this inverse association became less pronounced as DDAH1 increased on the multiplicative odds scale. It would therefore be inappropriate to conclude simply that higher DDAH1 amplified the protective association of arginine. Instead, the results indicate that the arginine-AD relationship differed across DDAH1 levels. One possibility is that elevated circulating DDAH1 in AD represents a compensatory response to impaired nitric oxide signaling, endothelial stress, increased ADMA, or altered substrate utilization. Under this model, higher DDAH1 could coexist with disease because the protein is upregulated in response to metabolic dysfunction without fully restoring normal pathway activity. Alternatively, circulating DDAH1 may reflect cellular injury, altered protein release, renal or vascular physiology, or other systemic processes rather than increased intracellular enzyme function. These competing explanations cannot be distinguished by the present cross-sectional analysis.

Experimental findings provide some support for a potentially beneficial role of arginine, but they also emphasize the need for caution in translating the present results into treatment recommendations. Oral arginine administration has been reported to reduce amyloid-β pathology and neuroinflammation in animal models of AD [11]. Such effects could involve improved nitric oxide signaling, but nitric oxide-independent mechanisms, including altered amino acid metabolism, immune-cell function, proteostasis, and protein aggregation, may also contribute. Moreover, an observational association in the UK Biobank does not establish that supplementation would benefit individuals with AD. Arginine metabolism is highly context-dependent, and increasing substrate availability could have different effects according to DDAH1 activity, ADMA concentration, arginase activity, renal function, vascular disease, and oxidative stress [14,15]. Clinical intervention would therefore require mechanistic stratification and prospective testing rather than supplementation based solely on circulating arginine concentration.

The persistence of the DDAH1-arginine interaction after adjustment for APOE ε4 is noteworthy. APOE ε4 showed the expected strong dose-related association with AD, while educational attainment was inversely associated with disease, consistent with its frequently proposed role as an indicator of cognitive reserve [16]. The continued association of the metabolic interaction after accounting for these variables suggests that the DDAH1-arginine axis may capture information not explained by established inherited susceptibility or educational exposure. This does not establish that the pathway is independent in a causal sense, because APOE may also influence lipid metabolism, inflammation, vascular function, and nitric oxide signaling. Nevertheless, the findings support the possibility that metabolic and endothelial pathways contribute to heterogeneity in AD risk within APOE strata and warrant evaluation alongside conventional genetic risk markers.

The neuroimaging findings were less consistent than the disease associations. DDAH1 was not significantly associated with most of the examined macrostructural measures, including the parahippocampal region, entorhinal cortex, amygdala, and deep white matter hyperintensity volume. In the hippocampal analysis, higher DDAH1 was associated with modestly lower total hippocampal volume, whereas arginine and the DDAH1-arginine interaction were not significant. This result does not provide structural confirmation of the interaction observed for AD diagnosis. Several factors may explain the discrepancy. The imaging sample was substantially smaller than the main proteomic cohort, reducing statistical power; the analyses were cross-sectional; and conventional regional volumes may not detect early endothelial, microvascular, synaptic, or metabolic abnormalities. DDAH1-related effects may be more closely reflected by cerebral perfusion, blood-brain barrier integrity, diffusion measures, vascular reactivity, or functional connectivity than by gross regional volume. The nominal DDAH1-hippocampal association should consequently be regarded as exploratory, particularly because multiple imaging phenotypes were examined and no correction for multiple comparisons was applied.

Taken together, these results place the DDAH1-arginine axis within a broader model of AD in which vascular, metabolic, inflammatory, and genetic factors interact rather than acting independently. The study extends previous arginine-metabolomic observations by integrating circulating DDAH1, arginine, APOE genotype, education, population structure, and neuroimaging in a single cohort. At the same time, the results do not establish temporal sequence or causality and do not justify describing DDAH1 or arginine as therapeutic targets without additional evidence. Longitudinal studies should determine whether these biomarkers precede incident AD, change during disease progression, or reflect consequences of established disease. Replication should include direct measurements of ADMA and nitric oxide bioavailability, the assessment of renal and vascular function, medication and dietary exposures, and evaluation in more diverse populations. Such studies could clarify whether the DDAH1-arginine interaction identifies a reproducible metabolic subtype of AD or primarily reflects compensatory systemic responses to neurodegeneration.

Limitations

Several limitations should be considered.

First, the study was observational and cross-sectional, preventing causal inference regarding the directionality of the observed associations. Elevated DDAH1 may represent a compensatory response to disease rather than a causal mediator of pathology.

Second, Alzheimer’s disease diagnoses in the UK Biobank were based on available clinical and registry-derived data rather than neuropathological confirmation, introducing the possibility of diagnostic misclassification. The relatively small number of AD cases compared to controls may also limit statistical precision for some subgroup analyses.

Third, circulating proteomic measurements may not fully reflect central nervous system biology. Plasma DDAH1 levels may differ from enzymatic activity within the brain microenvironment, and direct measurements of ADMA or nitric oxide bioavailability were not available.

Fourth, although we adjusted for major demographic and genetic covariates, residual confounding remains possible. Vascular comorbidities, medication use, renal function, diet, and inflammatory conditions could influence arginine metabolism and were not comprehensively modeled in the present analysis.

Fifth, the MRI analyses relied on macrostructural imaging phenotypes and may not capture microvascular dysfunction, white matter integrity, synaptic alterations, or functional network changes more directly linked to nitric oxide signaling. More sensitive imaging modalities, including diffusion imaging or functional MRI, may be required to detect such effects.

Sixth, the findings were derived from a predominantly middle-aged to older adult UK Biobank population and require external validation in independent and ethnically diverse cohorts.

Finally, selection bias is also possible because inclusion in the principal analyses required the availability of the relevant proteomic, metabolomic, genetic, and clinical variables, while imaging analyses required participation in the UK Biobank imaging program and the availability of the selected imaging-derived phenotypes. Complete-case analysis may therefore have selected participants who differ systematically from the broader UK Biobank cohort. In addition, renal function, vascular physiology, medication use, dietary factors, and other systemic determinants of arginine metabolism and DDAH1 biology could provide alternative explanations for some of the observed associations. The secondary biomarker and neuroimaging analyses were exploratory and were not adjusted for multiple comparisons, increasing the possibility of false-positive findings. These results, particularly the nominal hippocampal association, therefore require independent replication.

## Conclusions

In this UK Biobank analysis, DDAH1 showed the strongest case-control difference among the arginine-related proteins examined, and the interaction between DDAH1 and systemic arginine remained independently associated with AD status after adjustment for demographic, educational, and genetic factors. These findings identify the DDAH1-arginine axis as a metabolic correlate of AD and are consistent with, but do not establish, the involvement of altered arginine-nitric oxide signaling in AD biology. The largely negative macrostructural MRI findings indicate that the observed metabolic association does not translate straightforwardly into detectable regional structural changes. Longitudinal studies incorporating direct measurements of ADMA, nitric oxide pathway activity, renal and vascular determinants, and independent replication will be necessary to determine temporality, mechanism, and potential therapeutic relevance.
